# An Integrative Approach to Assessing the Impact of Mercury (Hg) on Avian Behaviour: From Molecule to Movement

**DOI:** 10.3390/jox15040117

**Published:** 2025-07-09

**Authors:** Dora Bjedov, Mirta Sudarić Bogojević, Jorge Bernal-Alviz, Goran Klobučar, Jean-Paul Bourdineaud, K. M. Aarif, Alma Mikuška

**Affiliations:** 1 Department of Biology, Josip Juraj Strossmayer University of Osijek, Cara Hadrijana 8/A, 31000 Osijek, Croatia; dora.bjedov@biologija.unios.hr (D.B.);; 2 Independent Researcher, St. 4A# 3S-81, Jamundí 764007, Colombia; 3 Department of Biology, Faculty of Science, University of Zagreb, Rooseveltov trg 6, 10000 Zagreb, Croatia; 4 CNRS, UMR 5234, European Institute of Chemistry and Biology, University of Bordeaux, 2 Rue Robert Escarpit, 33607 Pessac, France; 5 Centre for Environment and Marine Studies, Research & Innovation, King Fahd University of Petroleum & Minerals, Dhahran 31261, Saudi Arabia

**Keywords:** Tinbergen’s postulates, sublethal mercury toxicity, behavioural ecology, trophic transfer of mercury, wildlife conservation

## Abstract

Mercury (Hg) pollution is a widespread ecological threat with sublethal effects on wildlife. Birds, due to their ecological diversity and sensitivity, serve as effective models for evaluating the behavioural impacts of Hg exposure. This review applies Tinbergen’s four questions: causation, ontogeny, function, and evolution, as an integrative framework. Mechanistically, Hg disrupts neuroendocrine pathways, gene expression, immune function, and hormone regulation, leading to behavioural changes such as reduced foraging, altered parental care, and impaired predator avoidance. Early-life exposure affects neural development, learning, and social behaviour into adulthood. Functionally, these changes reduce fitness by compromising reproduction and survival. Phylogenetic comparisons show interspecific variability, with piscivorous and insectivorous birds exhibiting high Hg burdens and sensitivity, linked to ecological roles and exposure. Behavioural responses often precede physiological or demographic effects, highlighting their value as early indicators. Both field and laboratory studies show that even low Hg concentrations can alter behaviour, though outcomes vary by species, life stage, and exposure route. Integrating behavioural endpoints into ecotoxicological risk assessments is essential to improve conservation strategies and understanding of sublethal pollutant effects on wildlife.

## 1. Introduction

Mercury (Hg) is a significant global environmental pollutant, notable for its persistence, tendency for bioaccumulation, and recognised neurotoxic effects on wildlife, particularly avian species [1,2]. Mercury is emitted into the environment via natural geological processes [3] and increasingly through human activities, including fossil fuel combustion, industrial operations, and particularly artisanal and small-scale gold mining (ASGM) [1,4,5]. Artisanal and small-scale gold mining alone accounts for approximately 37% of global anthropogenic Hg emissions, while stationary combustion of coal contributes about 21% [5]. A review by Pfeiffer and Lacerda [6] estimated that gold mining in the Brazilian Amazon releases 30–150 t of Hg annually through amalgam combustion, leading to pollution of river sediments (0.30–3.00 μg·g^−1^, up to 19.80 μg·g^−1^), aquatic predators (>1 μg·g^−1^), and human hair in fish-eating populations (up to 70 μg·g^−1^). Consequently, pollutants not only affect immediate health but also compromise the overall reproductive health of populations, amplifying the potential for long-term ecological damage. By observing and analysing bird behaviour, it is possible to assess how pollutants of anthropogenic origin affect the health and viability of individuals, populations, and ecosystems [7,8], particularly during the breeding season, when pairs are easily observed and behavioural irregularities are more apparent.

Currently, Hg is considered a major worldwide pollutant due to its capacity to biomagnify, bioaccumulate, and remain persistent in ecosystems. Elemental Hg (Hg^0^) is the most well-known. Inorganic Hg, either as Hg^+^ (mercurous) or Hg^2+^ (mercuric), is commonly encountered in compounds such as HgS, HgCl, and Hg(OH), which can be associated with organic substances or suspended particles [9]. The divalent inorganic Hg associated with the dissolved organic matter (DOM) in freshwater systems is bound to thiols presented by the DOM and is found and characterised under two chemical structures: nanoparticles of ꞵ-HgS and dithiolates Hg(SR)_2_ [10]. Contrary to a strongly held belief, divalent inorganic Hg bound to DOM is bioavailable to fish that accumulate Hg as dithiolate and tetrathiolate complexes [10].

Due to the creation of covalent bonds, organic Hg compounds have been identified, such as the methylmercury ion (HgCH_3_^+^) and its neutral forms, which are methylmercury chloride (CH_3_HgCl) and methylmercury hydroxide (CH_3_HgOH), which are the most concerning species in the environment from an ecotoxicological perspective [9]. Other known organic mercurial compounds are dimethylmercury (DMHg), thimerosal, and phenylmercuryacetate. Phenylmercuryacetate is used as a fungicide, herbicide, and algicide, and thus can be ingested by animals, including granivorous and frugivorous birds; it is also found in antibiotic eye drops, eye cosmetics, shampoos, and paints [11]. Thimerosal, which is converted by liver cytochrome P450 into ethylmercury, is still being used as a preservative in some cosmetics, topical pharmaceuticals, contact lens preservative solution, and in paediatric vaccines [12], and is therefore not suspected to represent harm to birds. Dimethylmercury is by far the most toxic form of Hg [13,14]. DMHg is produced in deep waters in the oceans and seas and is the source of methylmercury (MeHg) through the demethylation process and upwelling transport to surface waters [15]. MeHg is the most toxic form after DMHg and accumulates in tissues of animals feeding in the aquatic food chain [16,17]. MeHg does not circulate freely in the blood and does not accumulate as free MeHg in animal tissues but rather forms L-cysteinate complexes, as was demonstrated with human serum albumin in blood and with keratins in hair [18]. This is the chemical structure also found in muscles of piscivorous fish and in breast feathers and brain of the Clark’s grebe (*Aechmophorus clarkii*) [19].

In both terrestrial and aquatic ecosystems, inorganic Hg can undergo microbial transformation into MeHg, an organometallic compound that exhibits high bioavailability and is biomagnified through food webs, resulting in elevated concentrations in organisms and especially top predators, including avian species [20,21]. Birds acquire Hg and MeHg primarily through their diet, with piscivorous species such as loons and grebes among the most heavily exposed due to the high MeHg content in aquatic prey [22]. This accumulation is accompanied by biomagnification within aquatic food webs, subsequently leading to adverse effects, as the rate of Hg intake exceeds the rate at which it can be excreted [23,24]. Ground-foraging insectivores and scavengers may also accumulate Hg through polluted terrestrial food sources [25]. Dosing trials and field investigations established toxicity limits and the consequences of Hg exposure in aquatic birds, whereas terrestrial species at lower trophic levels were neglected due to a perceived low potential for accumulating hazardous Hg concentrations [26]. Exposure to Hg has been shown to have a range of toxic effects in birds [2,27,28]. Birds are widely recognised as valuable bioindicators of environmental pollution due to their broad geographic distribution, ecological diversity, and sensitivity to pollutants [26,29,30,31,32,33]. Mercury increases in concentration through food webs via biomagnification [16]. In birds, Hg is primarily neurotoxic [34], though it can also disrupt other physiological systems, including endocrine and reproductive functions [22,30,35,36,37,38]. Non-destructive sampling techniques have facilitated the monitoring of Hg, as well as other heavy metal(loids), exposure in birds, including blood samples from coastal and pelagic birds [39,40], feather samples [41,42,43], and faecal samples from wading birds [44] and the pied flycatcher (*Ficedula hypoleuca*) [45]. These approaches not only support ethical research practices but also provide valuable insight into both short- and long-term pollutant exposure.

Traditionally, ecotoxicological research has primarily focused on evaluating the adverse effects of pollutants on physiological and reproductive endpoints in wildlife populations. However, there is a growing and crucial recognition of the pivotal role that animal behaviour plays in mediating the impacts of environmental stressors on an individual’s fitness and overall population dynamics [46]. Behaviour, as a sensitive and often rapid response to environmental cues, is intrinsically linked to an individual’s ability to survive, reproduce, and interact effectively with its surroundings, particularly when faced with pollution [47]. The study by Peterson et al. [48] emphasises the need to integrate behavioural ecology, toxicology, and conservation to better understand how pollutants, such as heavy metals, affect animal behaviour and ecological stability. Importantly, behavioural alterations can manifest at sublethal concentrations of pollutants, often preceding more overt physiological or reproductive impairments, thus serving as early warning signals of ecological disruption [28,47]. These seemingly subtle changes, such as alterations in foraging strategies [29], predator avoidance behaviours [36,49], and parental care [50,51], can have profound consequences for individual survival and, ultimately, population persistence [47,52], i.e., the ability of a population to maintain viable numbers and avoid extinction over time, despite environmental pressures [53]. Sublethal exposure to Hg in birds has been linked to fitness reductions. Existing evidence strongly indicates that Hg exposure negatively impacts reproductive output, suppresses immune function, and leads birds to avoid energetically demanding behaviours. However, for other endpoints, such as specific measures of reproductive success, endocrine function, and body condition, findings remain inconsistent, highlighting the need for further investigation; evidence of adverse effects is weaker or contradictory, requiring further study [28,54].

This review aims to provide a comprehensive synthesis of existing knowledge on the behavioural ecotoxicology of Hg in birds, using Tinbergen’s four questions, causation (mechanism), ontogeny (development), function (adaptive value), and evolution (phylogeny), as a conceptual framework [55]. This approach enables systematic organisation and interpretation of the diverse effects of Hg exposure on bird behaviour, linking proximate mechanisms to ultimate evolutionary consequences and thereby reducing interpretive bias.

Defining target behaviours for study is crucial, especially since behaviour is a highly complex and dynamic process influenced by multiple factors, including genetic predisposition, environmental conditions, and prior experiences. Behavioural modifications can serve as early warning signs of potential population-level disruptions and are particularly valuable for understanding the long-term effects of environmental pollutants. Tinbergen’s four questions provide a comprehensive framework for studying animal behaviour, and their application in ecotoxicology is essential for evaluating the impact of environmental stressors such as pollutants on avian species. They guide researchers in investigating both the ultimate and proximate causes of behavioural changes, facilitating a structured approach to understanding the underlying processes in behavioural ecotoxicology. By exploring mechanisms of behaviour at multiple levels, genetic, biochemical, and physiological, this framework helps identify the causal pathways through which pollutant exposure affects wildlife. In the context of behavioural ecotoxicology, it is important to understand how pollutants such as Hg interfere with the physiological processes governing normal behaviour. Disruptions caused by pollutants may not only affect immediate behaviours, such as foraging or mating, but also lead to long-term alterations in neuroendocrine and immune systems, further impacting individual fitness and survival [48].

Accordingly, we grouped research according to the Tinbergen question they address: causation (mechanism): studies examining how Hg disrupts physiological, neurological, or hormonal pathways underlying behaviour, including impacts on brain development, neurotransmitter systems, cognitive performance, and hormonal regulation. Ontogeny (development): research focused on how early-life Hg exposure, including maternal transfer to eggs or juvenile exposure, influences the development of behavioural traits across life stages. Function (adaptive value): research investigating how Hg-induced behavioural changes affect survival and reproductive success, such as foraging efficiency, predator avoidance, mate choice, and parental care, which ultimately influence population dynamics. Evolution (phylogeny): comparative and evolutionary studies exploring interspecific variation in Hg sensitivity, detoxification mechanisms, and life-history traits, helping to identify broader patterns and constraints in extrapolating findings across taxa. By explicitly applying this framework, we not only catalogue documented behavioural effects of Hg but also place them in an integrative evolutionary–ecological context. This approach highlights gaps in current knowledge and suggests priorities for future research to inform effective conservation and management strategies in Hg-polluted environments.

## 2. Mercury in the Environment and Avian Exposure

### 2.1. Sources and Concentrations of Mercury in the Environment

Mercury is released from geological sources into the atmosphere through natural processes such as volcanic eruptions and tectonic activity [56,57]. Its natural geochemical cycle includes atmospheric dispersion, deposition in terrestrial and marine environments, and subsequent re-emission. Anthropogenic activities have significantly disrupted this cycle by reintroducing Hg from long-term geological storage into the atmospheric system, via coal combustion, mining, and industrial operations [57]. As a result of these anthropogenic activities, Hg is now more widely distributed and will persist in circulating in the atmosphere, oceans, and land for hundreds to thousands of years, posing risks to both ecosystems and public health [58]. Currently, China is the largest atmospheric source of Hg pollution in the world [59]. In addition to China, major contributors to global anthropogenic Hg emissions include India, Indonesia, Russia, Brazil, and Peru, primarily due to coal combustion, ASGM, and industrial processes. These countries, along with China, account for a significant share of the estimated 2220 tonnes of Hg released to the atmosphere annually from human activities [60]. There has been a consistent 3–5-fold increase in Hg deposition in other parts of the world, such as boreal and Arctic regions [61,62,63], as well as continental North American regions [64]. It has been attributed to enhanced atmospheric transport of anthropogenic Hg emissions from lower latitudes (via prevailing circulation patterns), intensified wildfires in boreal forests releasing stored Hg, and climate-driven processes such as permafrost thaw and increased methylation in warming soils and wetlands [65,66,67,68].

In unpolluted, background environments, total Hg levels in soil typically range from 0.07 to 0.22 mg kg^−1^, with MeHg concentrations between 0.30 and 3.50 µg·kg^−1^ (mean ~1.60 µg·kg^−1^), generally representing less than 1% of total Hg [69]. In contrast, Hg-polluted regions, e.g., mining areas, can exhibit soil total Hg concentrations exceeding 300 mg·kg^−1^ and MeHg levels above 20 µg·kg^−1^. Correspondingly, MeHg in rice grains remains below 4 ng·g^−1^ in unpolluted areas but can reach up to 18 ng·g^−1^ in Hg mining regions [70]. Paddy soils, waterlogged, anaerobic soil characteristic of flooded rice fields, are of particular concern, as their redox and microbial conditions promote high Hg methylation potential, leading to MeHg accumulation in rice at levels up to two to three times greater than in other edible plants [71]. In vegetation, total Hg concentrations vary widely depending on Hg sources and pathways. In background areas, aboveground plant tissues typically contain less than 100 ng·g^−1^ total Hg (dry weight), with leaves averaging around 26 µg·kg^−1^ and less than 1% in methylated form. These levels mainly result from both soil and atmospheric uptake through plant stomata of Hg^2+^. In polluted areas, plant total Hg can exceed 1000 ng·g^−1^, particularly in leaves, due to direct atmospheric deposition [72].

### 2.2. Birds as Bioindicators and Their Susceptibility

Birds have long been recognised as valuable indicator species due to their strong association with general environmental conditions. While there are still reservations regarding their ability to directly and rapidly influence ecosystem properties and the cascading effects on other taxa, the advantages of using birds as indicators remain significant [33,73]. Birds are easy to detect and observe in their natural habitats, often signalling their presence through vocalisations, vivid plumage, and diurnal activity patterns [74]. Their taxonomy is well-established, and many species can be readily identified in the field [73]. Birds are also widely distributed, occupying a broad range of habitats and ecological niches [74]. Moreover, their distribution, abundance, habitat preferences, biology, and life histories are well-documented [73]. Positioned near the top of the food chain, birds are particularly sensitive to ecological disturbances and the accumulation of environmental pollutants [74,75]. Many bird species play essential roles in pollination and seed dispersal, contributing to the maintenance and resilience of ecosystems [74].

An additional aspect of birds as effective bioindicators is their sensitivity to environmental Hg [76]. Recently, studies have shown songbirds accumulate higher Hg concentrations than previously assumed. This includes terrestrial insectivorous songbirds whose habitat is not in proximity to any known present or past point-source pollution [77,78]. Controlled dosing experiments and field research involving songbirds have lately revealed that, at the upper end of environmentally observed levels, Hg can cause many of the same detrimental effects observed in piscivorous aquatic birds and other non-passerines, including disrupted endocrine and immune system function, behavioural disturbances, and altered reproductive success [28].

When studying species of conservation priority, it is crucial to employ non-destructive and minimally invasive sampling strategies to minimise stress and uphold ethical standards. Mercury can be measured in various bird tissues using both invasive and non-invasive methods, each with trade-offs in accuracy and the type of exposure assessed. Non-invasive samples, e.g., shed feathers, regurgitated pellets, or unhatched eggs, are advantageous for ethical and logistical reasons, though they may be less precise than invasive tissues due to environmental pollution or external deposition [79]. Blood and its fractions are especially effective for tracking environmental Hg and offer relatively high accuracy in reflecting recent, acute exposure [80,81,82,83]. For this reason, blood sampling remains a dependable method for determining Hg levels across diverse populations and is suitable for broad-scale assessments [30]. Feathers, while widely used due to their ease of collection and ability to reflect cumulative Hg burden, primarily indicate chronic exposure over the period of feather growth and may not reflect recent environmental Hg levels [42]. For example, Goutner et al. [84] observed that feather Hg content tends to rise with age. Furthermore, eggs have been used to explore the maternal transmission of Hg from females to their young [80]. Internal organs such as liver and kidneys, accessible only through invasive sampling or post-mortem, provide the highest resolution for long-term and accumulated Hg exposure due to their roles in storage and detoxification. Piedra et al. [85] analysed Hg in liver, kidney, and muscle tissues in white storks (*Ciconia ciconia*). Such findings highlight the value of organ-focused assessments for understanding prolonged exposure to Hg, as these tissues are central to Hg storage and detoxification. In summary, blood is more accurate for assessing acute, recent exposure, while feathers and internal organs better represent long-term, chronic accumulation, with internal tissues offering the highest accuracy where ethical considerations allow.

### 2.3. Routes of Exposure

Mercury enters birds primarily through trophic transfer, meaning Hg exposure occurs through multiple dietary routes, with the highest risk associated with fish consumption. Monitoring Hg concentrations across various trophic levels is essential for understanding exposure risk and ecological impact [86,87]. The key sources include polluted fish, small mammals, insects, and, to a lesser extent, plant matter such as seeds [2,88].

In aquatic systems, predatory fish species at the top of the food chain can have Hg concentrations ranging from 0.30 to over 1.50 mg·kg^−1^ wet weight, depending on age, size, and local pollution levels. Experimental studies in Atlantic salmon (*Salmo salar*) have shown that MeHg accumulates much more readily in internal organs and muscle than inorganic Hg, with assimilation rates of 23% versus 6%, respectively. Notably, fish fed just 5 mg·kg^−1^ dry weight of MeHg exceeded current food safety limits and exhibited biological signs of toxicity, such as altered blood haematology and increased liver metallothionein levels [89]. These high levels pose a significant risk to piscivorous birds.

Field mice in polluted areas can accumulate Hg through dietary intake and incidental soil ingestion. Mercury from the soil is absorbed by plants, which are consumed by small mammals, leading to bioaccumulation in their tissues. Published Hg concentrations in field mice range from 0.05 to 0.50 mg·kg^−1^ wet weight, with higher levels observed near pollution hotspots, such as those exceeding 5 mg·kg^−1^ in soils around the Rio Grande, a river in Illinois and New Mexico, USA [90,91]. Predatory birds that consume small mammals are exposed to Hg through the ingestion of the entire body of their prey, including organs like the liver and kidneys, where Hg tends to concentrate. Even if Hg levels in the brain of the prey are low, birds can still be significantly exposed to Hg through cumulative dietary intake over time. As these birds feed on multiple rodents over time, the Hg in their prey can accumulate, potentially reaching harmful levels.

Insects, especially those inhabiting polluted terrestrial environments near Hg mining areas, can serve as sources of Hg exposure. According to data from various insect trophic guilds in Mexico, Hg concentrations in insect bodies vary widely, with herbivorous insects containing between 0.19 and 106.10 mg·kg^−1^, decomposers ranging from 0.76 to 21.98 mg·kg^−1^, and predaceous insects from 0.57 to 12.95 mg·kg^−1^ wet weight [92]. Consequently, insectivorous birds may be exposed to significant Hg levels via their diet.

Although plants generally exhibit lower Hg concentrations compared to other organisms, they can still act as notable sources of exposure, particularly when grown in Hg-polluted soils. In regions of Uganda polluted by clandestine gold mining, it has been reported that total Hg levels in wild plant species can vary drastically, from as low as 0.03 mg·kg^−1^ in *Hyptis suaveolens* to over 868 mg·kg^−1^ in *Guizotia scabra*. While species like *Cynodon dactylon* and *Datura stramonium* show relatively low concentrations, 0.03 and 0.17 mg·kg^−1^, respectively, others such as *Fuirena umbellata*, *Cyperus articulatus*, and *Typha capensis* can accumulate much higher levels, exceeding 70 mg·kg^−1^ [93]. In China, cultivated grains from areas surrounded by coal-fired power plants have also shown variations in Hg levels, with rice containing between 24.99  ±  1.99 and 62.95  ±  3.88 mg·kg^−1^, and maize ranging from 0.55  ±  0.63 to 21.18  ±  0.67 mg·kg^−1^ [94]. These findings suggest that even vegetation, depending on species and location, may pose a chronic dietary Hg risk to herbivorous and granivorous birds.

## 3. Behavioural Effects of Mercury on Birds

To interpret the diverse behavioural effects of mercury exposure in birds, we organised our synthesis according to Tinbergen’s four questions. This framework provides a structured lens through which we examine proximate mechanisms, developmental patterns, fitness implications, and evolutionary perspectives, thereby offering an integrative understanding of how Hg pollution influences avian behaviour [48].

Molecular changes resulting from exposure to pollutants, such as alterations in gene expression and protein synthesis, can manifest as behavioural shifts. These molecular responses are highly interconnected, and studying them in parallel with behavioural changes provides deeper insight into the complex interactions between pollutants and their effects on wildlife [95]. For example, changes in the expression of genes related to stress response or detoxification enzymes may be linked to observable shifts in animal behaviour, including reduced reproductive success or altered migration patterns, which may affect species persistence, i.e., their survival/viability over time despite the environmental challenges [53]. Regarding altered migration patterns, one key mechanism involves disruption of lipid metabolism critical for sustained flight. Mercury, particularly in the form of MeHg, alters the expression of peroxisome proliferator-activated receptors (PPARs), which regulate mitochondrial fatty acid oxidation, an essential process for powering migratory endurance flights [96,97,98]. Concurrently, Hg exposure reduces haemoglobin levels by inhibiting heme synthesis, thereby limiting oxygen delivery to working muscles during flight [99,100]. These effects constrain aerobic capacity and can compromise the ability of birds to complete long-distance migrations [101,102]. Importantly, Hg also interferes with neuroendocrine stress regulation by disrupting the hypothalamic-pituitary-adrenal (HPA) axis and altering corticosterone signalling, which are essential for energy mobilization and behavioural responses during migration [102,103,104]. Together, these disruptions can delay migration, reduce stopover refuelling efficiency, impair arrival condition, and ultimately affect survival and reproductive success. However, the impact of pollutants may not always be negative; adaptive responses, such as behavioural plasticity or genetic resistance to specific pollutants, can lead to evolutionary shifts in populations, as seen in some avian species chronically exposed to heavy metals or pesticides [48].

### 3.1. Mechanism (Causation): How Does Mercury Affect Bird Behaviour?

**Figure 1:**
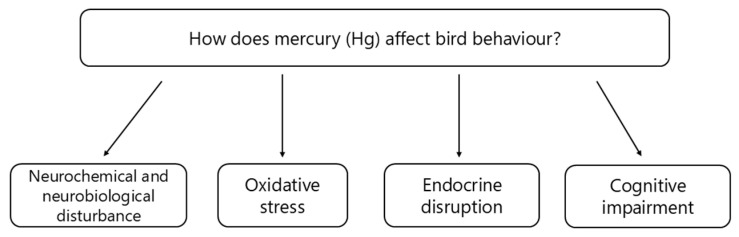
Schematic representation of how mercury (Hg) disrupts homeostasis, subsequently affecting behaviour in birds.

There is a growing recognition of Hg impact on bird behaviour [28,105], primarily as neurochemical and neurological disturbance, oxidative stress, neurotransmitter dysfunction, endocrine disruption, and cognitive impairment (Figure 1). The interaction between Hg and neuroreceptors, such as the blockade of muscarinic acetylcholine receptors (mACh), has implications for synaptic transmission and overall neural communication, which are essential for normal behavioural responses [106]. On a neurophysiological level, MeHg directly affects certain neurotransmitter receptors, mainly mACh receptors and N-methyl D-aspartate (NMDA) receptors [106,107]. The decreased sensitivity of NMDA receptors additionally exacerbates these effects, impairing excitatory neurotransmission and neural plasticity [107]. The ability of MeHg to cross the blood-brain barrier introduces additional complications by promoting oxidative stress in neurons and accumulating in the brain, essential for behavioural regulation [28,108]. The increased production of reactive oxygen species (ROS), as a consequence of Hg accumulation, has detrimental effects on neuronal integrity by disrupting mitochondrial function and impeding essential ATP production [109,110]. Henry et al. [111] confirmed oxidative stress mechanisms in songbirds exposed to dietary MeHg, supporting the idea that this pathway is important in Hg toxicity. The reduction in glutathione (GSH) levels, due to the binding of Hg to sulfhydryl (-SH) groups, severely limits the cell’s capacity for detoxification and antioxidant defences, further amplifying oxidative stress and cellular damage.

Neurotransmitters, such as dopamine and serotonin, are crucial for modulating avian behaviour. Disruption in signalling affects key regulators of motivation, social interactions, learning processes, and foraging behaviours [28]. Van den Brink et al. [112] documented specific neurochemical changes in Arctic barnacle goslings (*Branta leucopsis*). Namely, elevated levels of Hg were positively associated with an increase in dopamine D2 receptors in the brain, but there was no such relationship for NMDA receptors, suggesting a specific, subtle neurochemical alteration rather than broad neurotoxicity [112]. Reduced NMDA receptor binding was associated with Hg concentrations in wild bald eagles (*Haliaeetus leucocephalus*) and common loons (*Gavia immer*) [107,113]. Conversely, no variation in NMDA receptor affinity was identified in thick-billed murres (*Uria lomvia*), Arctic terns (*Sterna paradisaea*), or herring gulls (*Larus argentatus*) [114,115]. Studies on domestic poultry revealed that in ovo Hg exposure (MeHg-chloride) did not alter NMDA receptor binding in quail or chickens in one trial, though another using MeHg-cysteine found increased receptor binding in chickens [116]. It is important to consider the chemical form of MeHg, as its effects, such as alterations in NMDA receptor binding, appear to depend on the binding compound used (e.g., MeHg-chloride vs. MeHg-cysteine) [117]. Indeed, it has been shown that in mice exposed to MeHg through diet for 55 days, the spontaneous alternative behaviour in a Y-shaped maze was decreased only for mice fed the fish flesh-associated MeHg as compared to those fed MeHg-chloride added to diet [118]. The matrix in which MeHg is dissolved is also important, since when mallard (*Anas platyrhynchos*) eggs were injected with MeHg-chloride dissolved in corn oil or water, 16% of the eggs injected with 1.60 µg·g^−1^ Hg dissolved in water hatched, which was statistically lower than the 37% hatch rate of eggs injected with the same concentration of MeHg-chloride dissolved in corn oil [119].

Furthermore, research on zebra finches (*Taeniopygia guttata*) revealed receptor-level changes linked to cognitive and locomotor impairments following prolonged exposure to dietary MeHg [120]. Sub-lethal, environmentally relevant Hg exposure was found to selectively impair spatial memory, increase activity levels, and reduce social dominance, indicating targeted neurological disruption that may compromise foraging efficiency, social hierarchy, and overall fitness [120]. Elevated MeHg levels disrupt the levels of reproductive hormones like oestradiol, testosterone, follicle-stimulating hormone (FSH), and luteinizing hormone (LH) in the blood. Frederick and Jayasena [50] observed altered pairing behaviour and reduced reproductive success in white ibises (*Eudocimus albus*) exposed to environmentally relevant dietary MeHg concentrations, 0.05–0.30 mg·kg^−1^, potentially linking endocrine disruption with impaired mate selection or pair bond maintenance. Furthermore, analysis of faecal corticosterone concentrations was performed between the control and MeHg-exposed white ibises (to dietary MeHg of 0.05, 0.10, and 0.30 mg·kg^−1^), and the results suggest the pattern of nonlinear dose-response for MeHg [121]. The research by Adams et al. [121] suggests that endocrine effects of MeHg exposure could affect multiple endpoints in a nonlinear manner. Further expounding on the fact that Hg affects corticosterone, the HPA axis is altered as well and changes the release of corticosterone, the main glucocorticoid in birds that plays a role in stress responses [122,123]. Franceschini et al. [122] found that Hg exposure and the corticosterone response were complicated and depended on the stage of life of free-living tree swallows (*Tachycineta bicolour*). Moore et al. [124] described reduced stress responsiveness in zebra finches exposed to Hg. Mercury exposure also disrupts the secretion of prolactin, a hormone essential for the parental care behaviours of birds [125]. In Arctic seabirds, Tartu et al. [51,126] found that Hg-induced endocrine disruption could directly compromise parental investment by demonstrating associations between Hg levels, altered prolactin secretion, and increased egg neglect behaviours.

Methylmercury exposure is known to induce axonal degradation and various neurological abnormalities. In one juvenile saltmarsh sparrow (*Ammodramus caudacutus*), opportunistically sampled from a population with the highest mean blood Hg concentrations (1.50  ±  0.32 µg·g^−1^) among 25 breeding sites in the northeastern United States, pronounced cerebellar abnormalities were observed, including mispositioned Purkinje neurons across all three cerebellar layers and the persistence of an external granule cell layer—an embryonic structure typically absent post-fledging. Feather Hg concentrations in this individual measured 3.40 µg·g^−1^ in the first primary and 2.90 µg·g^−1^ in other flight feathers, and while no brain Hg levels were available, these findings suggest in ovo MeHg exposure from the maternal diet may have induced neurodevelopmental disruption [127], paralleling cerebellar malformations seen in human cases of foetal Minamata disease [128]. While this observation on the juvenile saltmarsh sparrow is based on a single individual and should be interpreted with caution, it may provide preliminary insight. The recovery of this saltmarsh sparrow is a rare event, as juvenile saltmarsh sparrows are likely highly vulnerable to predation and may go undetected in the field. Further sampling would be necessary to evaluate whether this observation reflects a rare ecological event or simply low detectability. In experimental studies, adult mallards administered dietary MeHg experienced axonal breakdown [129], while exposed mallard nestlings displayed both myelin loss and reduced neuron size [130]. Similarly, rock pigeons (*Columba livia*) experienced demyelination, though unlike mallards, their neurons became enlarged [131]. Double-crested cormorants (*Nannopterum auritum*) displayed damaged axons and enlarged myelin sheaths under similar treatment conditions [132]. MeHg-treated zebra finches demonstrated auditory dysfunction, evidenced by elevated brainstem response thresholds, lower response amplitudes, and prolonged neural latency to acoustic stimuli [133]. In another study, male zebra finches exposed in ovo to up to 3.20 mg·kg^−1^ egg MeHg exhibited increased telencephalon volume; however, overall brain mass and key song control nuclei, area X, the robust nucleus of the arcopallium, and high vocal centre (HVC) remained unaffected [134]. Research on raptor response to MeHg exposure was evaluated as well. American kestrels (*Falco sparverius*) exposed to dietary concentrations of MeHg exceeding 5 μg·g^−1^ presented with axonal deterioration and brain lesions [135]. Similarly, red-tailed hawks (*Buteo jamaicensis*) showed signs of axonal pathology following exposure to MeHg levels up to 5.20 μg·g^−1^ [136].

Furthermore, Hg exposure may impair critical enzymes like glutamine synthetase (GS), which plays a vital role in ammonia detoxification, regulation of excitatory neurotransmission via the glutamate–glutamine cycle, and antioxidant defence [137]. Glutamine synthetase did not change in MeHg-dosed chicken nestlings up to a dietary concentration of 6.40 μg·g^−1^, while no change in GS activity was found in older nestlings at any concentration [116]. In free-living bald eagles, there was a positive correlation between Hg and GS activity [113]. Glutamic acid decarboxylase activities have been found to either increase or remain the same in chickens and decrease in quail with in ovo administration of Hg [116] and were negatively correlated with inorganic Hg in bald eagles [113]. Gamma-aminobutyric acid either showed no change for chickens or quail, increased in chickens exposed to 6.40 μg·g^−1^ MeHg injected in egg, or decreased in chickens exposed to 3.20–6.40 μg·g^−1^ MeHg-cysteine [116]. Muscarinic cholinergic receptor density was unchanged in thick-billed murres and Arctic tern’s eggs dosed with MeHg of 0.05, 0.10, 0.20, 0.40, 0.80, 1.60, 3.20, and 6.40 μg·g^−1^ [115]. Research on herring gulls showed no changes in mACh receptor as well, in addition to a lack of effect on nicotinic cholinergic receptor density or nicotinic receptor alpha-7 mRNA expression [114]. Scheuhammer et al. [138] observed in common loons and bald eagles that liver Hg concentrations ranged widely, with substantial MeHg demethylation and co-accumulation of selenium (Se), while in the brain, bald eagles showed greater demethylation capacity than common loons, who retained more MeHg. However, both species exhibited similar neurochemical responses, including increased muscarinic receptor levels and decreased NMDA receptor levels with rising brain Hg concentrations [138]. Interestingly, the activities of cholinesterase (ChE) and monoamine oxidase (MAO) were not associated with Hg levels in either species [138]. In another sample population of common loons sampled after having died of botulism, no associations were observed between total Hg concentrations in brain and NMDA, and mACh receptor densities, as well as for MAO or ChE activities; however, common loons had relatively low Hg tissue concentrations and a molar excess of Se in their brain tissue, which is known for mitigating the impact of Hg [139].

Mercury exposure has also been shown to affect learning in songbirds. This impaired song development may have further implications for reproductive success and social interaction, which are heavily reliant on communication. Carolina wrens (*Thryothorus ludovicianus*), house wrens (*Troglodytes aedon*), Nelson’s sparrows (*Ammospiza nelsoni*), and song sparrows (*Melospiza melodia*) exposed to Hg pollution were impacted in their song behaviour. Songs recorded from the birds living on polluted sites were found to contain a lower diversity of notes and were sung at lower frequencies compared to those from birds on a reference site [140]. While the effects of total Hg on bird song are species-specific, exposure is associated with alterations, including lower-frequency, less complex songs, as well as faster songs with higher maximum frequencies and shorter intervals between bouts [141,142]. These findings align with similar research indicating that Hg interferes with the neural processes involved in learning, memory, and other cognitive functions [120,143]. On the other hand, the exposure via the injection of Hg in ovo did not alter the song of the zebra finch [134].

For example, great egrets (*Ardea alba*) showed reduced activity [144] and loss of motor coordination [100], while zebra finches became lethargic and struggled with balance and perching [145]. Following exposure to 13–27 mg·kg^−1^ MeHg, domestic pigeons displayed ataxia and impaired feeding accuracy, along with slower and less frequent responses in operant conditioning tests [131,146]. Feeding accuracy refers to an animal’s ability to precisely locate, target, and ingest food items using coordinated motor and sensory functions. In birds, reduced feeding accuracy often manifests as difficulty pecking at or grasping food, leading to longer handling times, missed strikes, or decreased food intake [147,148]. In American kestrels, motor impairments emerged after 26 days of dietary exposure to 5.02 mg·kg^−1^ wet weight MeHg-chloride, and after 39–49 days of exposure, all American kestrels died, with levels in the liver measured at approximately 77 mg·kg^−1^ wet weight [135]. Beyond laboratory settings, field and semi-field studies suggest that Hg can alter ecological behaviours such as migration and foraging, though effects vary by species. For instance, snowy egret (*Egretta thula*; total blood Hg 1.47, 3.39, and 1.89 µg·g^−1^ wet weight) migration timing [149] and common eider (*Somateria mollissima*) arrival at breeding sites [150] appeared unaffected by total Hg. Common loons with higher Hg levels (total blood Hg 3.00 µg·g^−1^) showed reduced food consumption, spending less time foraging for both themselves and their nestlings [22]. However, they compensated for this decrease by increasing their diving frequency, potentially attempting to secure food in a more energy-intensive manner [151]. In keeping with this, reduced foraging efficiency and longer hunting durations were observed in Hg-dosed great egrets [100,144]. Interestingly, responses were not uniformly negative. Great egrets maintained performance comparable to controls at a low level of exposure (0.50 mg·kg^−1^ MeHg-chloride), despite exhibiting reduced appetite. On the other hand, great egrets exposed to 5 mg·kg^−1^ MeHg-chloride became severely ataxic [144]. Dosed zebra finches exhibited less efficient foraging strategies, including slower response times and increased latency between foraging attempts, which coincided with elevated tissue Hg concentrations. Namely, zebra finches that died had the highest mean Hg levels (liver: 73  ±  16 µg·g^−1^; kidney: 65  ±  13 µg·g^−1^; brain: 20  ±  5 µg·g^−1^ wet weight), followed by neurologically impaired individuals (liver: 43 µg·g^−1^; kidney: 55  ±  18 µg·g^−1^; brain: 20  ±  4 µg·g^−1^), while asymptomatic birds showed lower burdens (liver: 30.50 µg·g^−1^; kidney: 35.50  ±  9.60 µg·g^−1^; brain: 14.10  ±  3.10 µg·g^−1^), suggesting a dose-dependent relationship between Hg accumulation and behavioural dysfunction [145].

Regarding the effect on gene expression, exposure of laughing gull (*Leucophaeus atricilla*) hatchlings to environmentally relevant dietary MeHg doses of 0 (control), 0.50, 1.00, and 2.00 µg·g^−1^ wet weight led to significant changes in hepatic gene expression associated with oxidative stress, metal detoxification, and energy metabolism. At 0.50 and 1.00 µg·g^−1^ MeHg, key genes such as methionine adenosyl transferase 1 alpha (MAT1A), important for cysteine and glutathione synthesis, were upregulated in a dose-dependent manner, indicating enhanced antioxidant defences against MeHg-induced ROS [152]. Similarly, mitochondrial genes cytochrome c oxidase subunits I and II (COI, COII) showed increased expression at the 1.00 µg·g^−1^ dose, reflecting mitochondrial adaptation to reduce ROS production. Genes regulating iron homeostasis exhibited differential responses: transferrin (Tf) expression decreased at the 1.00 µg·g^−1^ dose, potentially limiting iron-mediated oxidative damage, while ferritin (FER) expression tended to increase, promoting iron storage and detoxification. Notably, total glutathione levels and oxidative stress biomarkers remained relatively stable across these exposures, suggesting effective compensatory mechanisms at sublethal MeHg doses. At the highest exposure of 2.00 µg·g^−1^ MeHg, gene expression patterns returned near control levels, coinciding with reduced survival rates, implying overwhelmed detoxification pathways [152]. Disruptions in oxidative stress and energy metabolism genes caused by mercury exposure can impair neural function, potentially leading to altered foraging behaviour, reduced learning ability, and compromised motor coordination in birds.

### 3.2. Ontogeny (Development): How Does Mercury Exposure Affect Bird Behaviour Across Life Stages?

**Figure 2:**
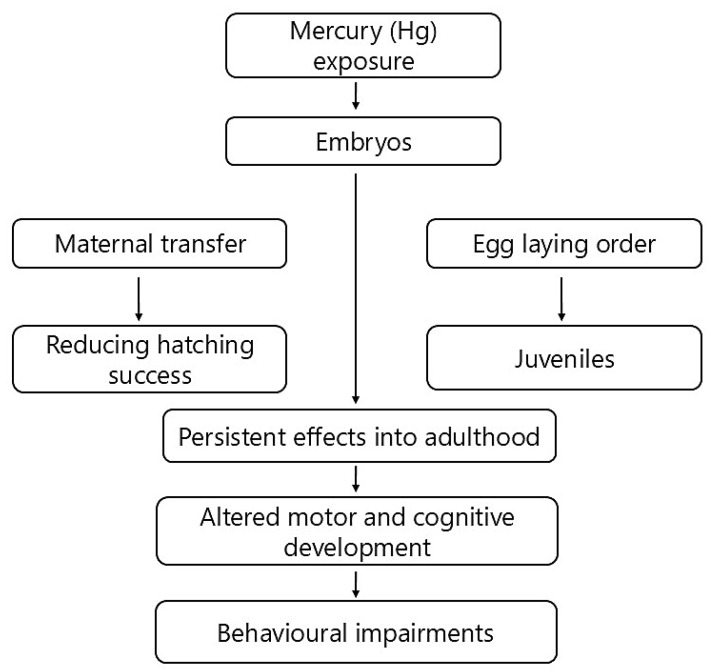
Scheme of how mercury (Hg) exposure during key developmental stages, embryonic and juvenile, affects bird behaviour later in life.

Bird developmental stages represent periods of heightened sensitivity to environmental stressors, during which Hg exposure can lead to behavioural impairments later in life [28,153] and references therein. Mercury exposure at critical developmental stages significantly shapes avian behavioural outcomes, with both embryonic and juvenile exposure routes playing critical roles (Figure 2).

Embryos are primarily exposed to Hg via maternal transfer, which occurs when females actively introduce Hg into the developing eggs [154,155]. The rate at which Hg moves from the mother’s body to the eggs varies according to the mother’s diet, body weight, the order in which the eggs are produced, and species-specific physiological differences [154,156]. Ackerman et al. [27] demonstrated that the quantity of Hg present in eggs is significantly affected by the order in which they are deposited, indicating that embryonic exposure can fluctuate within a clutch. Similarly, Ou et al. [157] discovered the laying sequence could influence the Hg concentration in the eggs of captive zebra finches, suggesting that there is variation in exposure within the brood. Evers et al. [158] found that although Hg concentration did not affect fertility, a decrease in egg volume was observed with increasing Hg levels, which may have implications for reproductive success in common loons. Further implications later in life, i.e., embryonic viability, may influence population-level behavioural dynamics as a result of increasing Hg levels in eggs, subsequently making it less likely that the eggs will hatch, and embryo malposition has been suggested as a possible reason for this [159,160].

Effects of Hg exposure during early development can persist into adulthood, even if subsequent Hg exposure diminishes [28]. Weiss et al. [161] described silent latency periods in MeHg poisoning, suggesting that neurological damage from early-life exposure might remain subtle or unnoticed until later life stages. Behaviour problems caused by hidden Hg effects might become more noticeable when there is a lot of mental, physical, or environmental stress, e.g., when animals are breeding or when there is a lot of competition.

Furthermore, under laboratory conditions, Hg has been linked to impaired motor skill development in hatchlings and fledglings [28], and Paris et al. [155] provided direct evidence demonstrating that zebra finches exposed to MeHg solely during embryonic and juvenile development exhibited significantly reduced cognitive performance in adulthood. Common loon nestlings with elevated Hg exposure spent less time back riding and more time preening, although no change was observed in their diving or swimming habits in lakes with increased total Hg levels in blood [162]. Additionally, experimental exposure to Hg in ovo led to various behavioural changes, such as faster platform crossing, increased time spent on platforms and in sunlight, and diminished reactions to parental calls and threatening stimuli [163,164]. The experimental observation suggests that exposure to Hg did not have an effect on the nestlings’ behaviour in response to hearing their mother’s calls [130,165,166] until the third generation of exposure, when they exhibited a decreased response [167]. On the other hand, when Hg was injected into white leghorn chicken eggs, the surviving nestlings did not differ in their response to a threatening stimulus; however, they took longer to right themselves [116]. For nestlings, the ability to right themselves is an important part of their motor development and survival skills. If they are placed on their back, they should be able to flip themselves over and stand up quickly, a behaviour that was altered after Hg exposure. These developmental abnormalities may persist into adulthood, manifesting behaviourally as impaired motor skills, diminished learning capacity, compromised spatial memory, and altered social behaviour as a result of early-life exposure [168]. Common loon nestlings exposed to MeHg exhibited altered growth rates, potentially affecting their subsequent behavioural performances related to foraging, predator avoidance, and territorial defence [22,158,169].

### 3.3. Function (Adaptation): How Do Mercury-Induced Behavioural Changes Affect Birds’ Survival and Reproduction?

**Figure 3:**
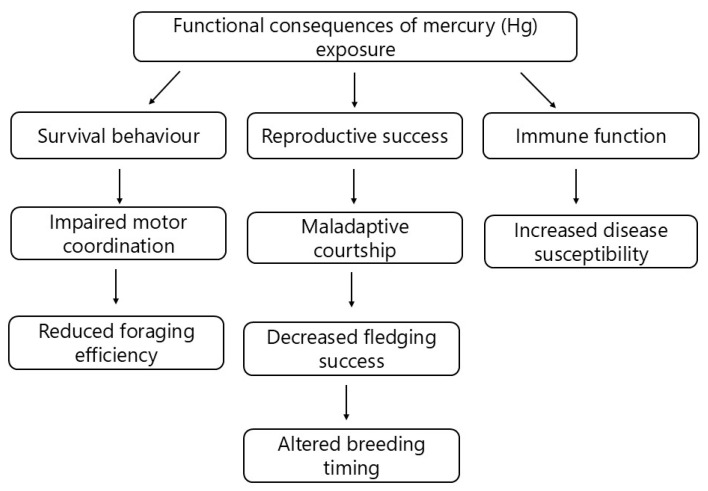
F Scheme of functional consequences of mercury (Hg)-induced behavioural alterations on bird survival and reproduction.

The functional component of Tinbergen’s four questions examines the adaptive significance of specific behaviours in relation to an animal’s reproductive success and survival [170]. In the context of Hg pollution, the functional consequences of Hg-induced behavioural alterations are evaluated by determining the extent to which sublethal Hg exposure affects the fitness and population stability of birds [28,153]. Although the behavioural shifts that result from Hg exposure may appear to be subtle, experimental studies have shown that these changes have significant implications for survival and reproductive success, thereby influencing the overall population dynamics (Figure 3) [36,120].

Several critical survival behaviours are adversely affected by Hg-induced behavioural changes, e.g., impairment of motor coordination and foraging efficiency, both of which are essential for food acquisition [28]. For instance, zebra finches that were exposed to dietary Hg exhibited cognitive deficits that substantially reduced their foraging success. Such changes could potentially result in a decrease in energy intake and an increase in the risk of starvation [120]. Kobiela et al. [36] discovered that zebra finches exposed to MeHg had a significant drop in body mass, particularly in high-predation settings, indicating altered foraging behaviour. Furthermore, Hg can reduce the efficacy of energy-intensive behaviours in aquatic birds, such as diving, by interfering with heme synthesis and impeding oxygen transmission [151]. MeHg exposure can significantly impair predator avoidance strategies by disrupting birds’ ability to perceive threats and respond appropriately [36,120,153]. Zebra finches, following exposure to Hg, exhibited maladaptive risk-taking behaviours, either excessively cautious or overly bold, both of which increased vulnerability and negatively affected survival. Specifically, the authors observed that zebra finches exposed to Hg took significantly longer to resume foraging after perceiving a predation threat, leading to substantial weight loss and illustrating the complex trade-off between the need to feed and the need to avoid predators [36].

Mercury exposure can have a significant impact on migratory performance, particularly at stopover sites. In migratory songbirds, Seewagen et al. [76,171,172] discovered correlations between impaired refuelling performance and elevated Hg concentrations. Refuelling performance refers to the ability of migratory birds to efficiently replenish energy reserves, primarily fat stores, during stopovers between migratory flights. Impaired refuelling performance, often caused by physiological or behavioural disruptions such as those induced by Hg exposure, can result in slower fat accumulation, extended stopover duration, or reduced migration speed and success [96,97,98,101,147,148]. This means that Hg exposure can significantly reduce survival during demanding long-distance migrations due to reduced physiological condition or impaired navigation abilities during stopovers. Seewagen et al. [173] investigated the effects of MeHg exposure on migration-related behaviour in birds. The authors dosed wild-caught yellow-rumped warblers (*Setophaga coronata*) with MeHg and tracked their spring migration. The exposed birds departed the release site significantly earlier than controls, suggesting that MeHg may influence migratory decision-making. However, MeHg did not appear to impair flight orientation.

The behavioural changes caused by Hg significantly disrupt numerous reproductive processes. For example, a reduction in reproductive success that affects pairing behaviours, companion choices, and courtship performance. The presence of Hg in the environment resulted in a decrease in the number of white ibises that were able to reproduce [50]. Exposure to Hg also influenced the reasoning, movement, and courtship of zebra finches. They were less adept at singing and responding to potential partners, which directly impacted their capacity to identify a mate and produce offspring [120,174]. Furthermore, Hg can affect nest-building behaviours, potentially leading to suboptimal nest quality, increased predation risk, and poor thermoregulation, which negatively affects offspring survival [175]. Although Chin et al. [175] found no Hg-induced effects on incubation behaviours in zebra finches, other studies indicate potential species-specific differences. Tartu et al. [51,126] observed increased egg neglect and altered breeding decisions in seabirds exposed to Hg, demonstrating indirect yet significant reproductive impacts. Parental care behaviours comprising offspring provisioning and survival, can be directly reduced as a consequence of impaired foraging efficiency due to Hg toxicity [28]. American kestrels fed a diet of meatballs containing 0.7 μg.g^−1^ dry weight of MeHg produced 24% fewer fledged juveniles than those consuming a control diet [176].

Continuous reduction in overall reproductive success is evident through lower clutch sizes, decreased hatching success, and reduced fledging success [50,177]. Varian-Ramos et al. [177] demonstrated that Hg-exposed zebra finches produced significantly fewer independent offspring annually due primarily to reduced fledging success. These adverse reproductive outcomes are made even stronger by the fact that embryo malposition has been linked to Hg-related hatching failures [159,178].

Mercury exposure can influence breeding timing, potentially disrupting the synchrony between reproductive events and optimal environmental conditions. For example, common loons with elevated blood Hg exhibited altered incubation and provisioning behaviours, including reduced nest attendance, lower feeding rates for chicks, and increased nest abandonment. Such behavioural disruptions during critical breeding periods negatively impact chick development, survival, and eventually population recruitment [22].

Although immune function is not a behavioural trait, Hg-induced immunosuppression indirectly affects survival-related behaviours [179,180,181]. Birds with compromised immune systems face increased susceptibility to diseases and parasites, potentially causing lethargy, decreased foraging activity, and heightened predation risk. Free-living tree swallows exposed to elevated Hg levels showed compromised immune competence, indirectly impacting their overall survival [180,181]. Furthermore, research on sexual selection in barn swallows (*Hirundo rustica*) has demonstrated that females select males with longer and more symmetrical tail feathers, traits that are positively associated with reproductive success and potentially indicative of genetic quality and immunocompetence. Experimental manipulations have shown that males with artificially elongated and symmetrical tails mated earlier and more successfully, suggesting that tail symmetry may act as an honest signal of an individual’s ability to withstand environmental stressors, including parasitic infestation. Increased tail asymmetry in males has been correlated with higher parasite loads, such as feather lice (*Mallophaga* sp.), which often feed on the white patches of tail feathers, leading to feather damage [182]. These findings imply a link between morphological symmetry, parasite resistance, and immune function. Emerging evidence suggests that exposure to Hg may further compromise immune responses and increase susceptibility to parasites, thereby indirectly influencing ornamental traits. For example, a recent study found that fluctuating asymmetry in rectrix feathers increased with Hg concentrations in Forster’s terns (*Sterna forsteri*), supporting the hypothesis that Hg can disrupt normal feather development [183]. Overall, adaptation in this context could be more related to physiological and genetic mechanisms (e.g., detoxification pathways), but behavioural adaptations to Hg pollution seem unlikely.

### 3.4. Evolution (Phylogeny): How Have Evolutionary Processes Shaped Species Differences in Responses to Mercury Exposure?

**Figure 4:**
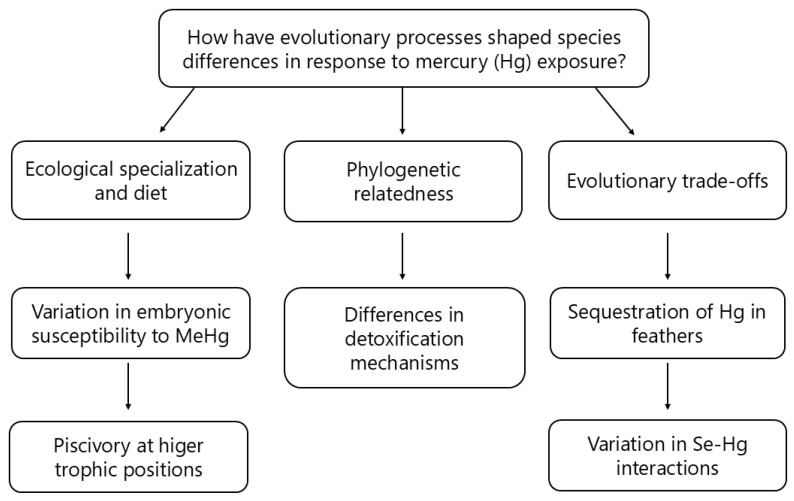
Schematic summary of evolutionary (phylogenetic) influences on species-specific responses to mercury (Hg) exposure in birds.

To comprehend the evolutionary (phylogenetic) aspect of how bird species handle Hg exposure, it is necessary to investigate the selective pressures that have influenced the physiological and behavioural adaptations in avian lineages. Tinbergen’s fourth question underlines the adaptive significance and evolutionary history of physiological and behavioural attributes [170]. This perspective encourages the investigation of whether specific bird species inherently possess characteristics influenced by their evolutionary history that affect their sensitivity and behavioural responses to Hg exposure. Pronounced variations in embryonic susceptibility to MeHg across species suggest evolutionary divergences driven by ecological specialization and diet [178,184]. Piscivorous species, occupying higher trophic positions, typically accumulate higher MeHg concentrations compared to insectivorous or granivorous species, resulting in differing selective pressures for detoxification or behavioural adaptations [2,29,153]. To strengthen the direct link to phylogeny, it is essential to emphasise how shared evolutionary histories between closely related species can influence their ability to detoxify Hg. Species within the same family or genus, such as the aforementioned zebra finch and house finch (*Haemorhous mexicanus*), may exhibit differences in their detoxification mechanisms, possibly due to evolutionary divergence shaped by distinct ecological pressures. However, it is important to clarify that the differences in detoxification abilities would likely be more strongly influenced by ecological factors (e.g., diet and habitat) and evolutionary trade-offs (e.g., specialisation vs. generalisation) than by their phylogenetic relatedness alone (Figure 4).

A crucial detoxification pathway involves sequestration of Hg in feathers, followed by its removal during moult. Birds that moult more frequently or extensively are better equipped to eliminate Hg from their bodies [29,185,186,187,188]. Additionally, Se–Hg interactions, where Se binds with Hg to reduce its toxicity, vary across species, likely due to differences in their diet and Se exposure [99,189]. In fact, Se does not directly protect animals against MeHg, since it has been found to be toxic in birds [190]. When the toxicities of both MeHg and selenomethionine were compared in three species (mallards, chickens, and double-crested cormorants), it appeared that, looking at the types of teratogenic events, selenomethionine alone caused more deformities than did MeHg alone [191]. A paradoxical and puzzling interaction effect was observed when MeHg was injected in combination with selenomethionine: the presence of MeHg resulted in less embryo mortality than had been seen with Se alone, but it increased the number of deformed embryos and hatchlings [192]. The protective role of Se against the deleterious effects of MeHg lies in its being embedded within the polypeptide chain of specific enzymes, more precisely selenoproteins. It has been found that in carnivorous fish liver, earthworms, and the kidney and liver of Clark’s grebe, selenoproteins are detoxify MeHg in a process combining demethylation and complexation by several Se residues of selenoproteins. In Clark’s grebe, Hg could be characterised under three different chemical forms: MeHg-cysteinate (MeHgCys), tetraselenocysteinate (Hg(Sec)_4_), and a dithiolate (Hg(SR)_2_). The contributions of these forms to the different tissues of those birds were as follows: in breast feathers and brain, 100% of MeHgCys, in muscles, 66% MeHgCys, 11% Hg(Sec)_4_, and 23% Hg(SR)_2_; in kidneys, 28% MeHgCys, 60% Hg(Sec)_4_, and 12% Hg(SR)_2_; and in liver, 14% MeHgCys and 86% Hg(Sec)_4_ [19]. Therefore, the detoxification process involving the intervening action of selenoproteins occurs in the kidneys and most efficiently in the liver.

Differences in species’ ability to handle hepatic demethylation processes, where toxic MeHg is converted to less harmful inorganic Hg, may reflect evolutionary adaptations to varying amounts of Hg in the diet [153,156]. Genetic polymorphisms affecting Hg accumulation, such as those found in zebra finches [193], may have evolved due to long-term exposure to Hg in specific ecological niches. Understanding how these genetic traits are passed on within populations could offer insights into how avian species might adapt genetically to Hg pollution over time. Applying ecogenomics could also reveal the underlying genetic mechanisms conferring Hg resistance, further supporting the process of evolutionary adaptation in response to environmental pollutants [194].

There is some evidence, both anecdotal and experimental, that birds exposed to environments with toxic prey, such as harmful algal blooms, develop learned behaviours to avoid consuming deadly toxins. Birds that have previously encountered toxic algae appear to recognise and avoid toxic prey, whereas naïve individuals, those without prior exposure, are at greater risk of intoxication. Additionally, feeding behaviour influences vulnerability: species that consume prey whole without tasting or sampling may be more susceptible to poisoning compared to those that selectively sample or reject unpalatable items. This learned avoidance likely acts as an important behavioural adaptation to reduce exposure to environmental toxins [195]. Although it is theoretically plausible that birds exposed to Hg may have developed strategies to avoid prey polluted with Hg or preferentially nest in areas with lower Hg exposure, empirical evidence supporting these behavioural adaptations is non-existent to our knowledge. Future research focused on the behavioural ecology of Hg-exposed populations is necessary to confirm whether these strategies exist and are linked to phylogenetic divergence [28].

## 4. Empirical Insights into the Behavioural Impacts of Mercury Exposure

Empirical research on Hg toxicity in birds reveals a complex interplay between species-specific sensitivity, tissue type sampled, and exposure context (laboratory vs. field). When examined collectively, the studies show that sublethal behavioural effects of Hg are widespread, but their manifestation is highly variable across taxa and experimental conditions.

### 4.1. Species-Specific Sensitivity

A key insight from the literature is that avian species differ considerably in their vulnerability to behavioural disruption by Hg (Table 1). Aquatic species such as the common loon appear especially sensitive, with multiple studies showing altered behaviour at both moderate and high blood Hg levels. The common loon exhibited increased lethargy and reduced incubation time at blood concentrations of 3.00 µg·g^−1^, and aberrant behaviours such as neglect of incubation and asymmetrical feather growth at levels over 4.10 µg·g^−1^ and 40 µg·g^−1^ in feathers, respectively [22]. Meanwhile, waterfowl, e.g., the mallard, displayed effects only at relatively high doses (0.50 µg·g^−1^ [167]), again highlighting interspecies differences in dose thresholds for behavioural disturbance. These findings suggest that fish-eating species may be more susceptible due to bioaccumulation through trophic transfer. However, responses appear more variable in passerines. Particularly, they showed no observable reproductive impairment at low Hg concentrations (from ~0.71 up to 2.54 µg·g^−1^ in blood [196]), indicating a degree of tolerance or resilience. Yet this is not consistent across the group; the tree swallow exhibited significantly reduced reproductive success and altered incubation behaviour at similarly low levels (e.g., 3.03 µg·g^−1^ in blood, 0.53 µg·g^−1^ in eggs [184,197]). This variation suggests that life history traits, diet, and metabolic processes likely mediate Hg sensitivity in passerines. Laboratory studies reinforce this variability. In the zebra finch, even low dietary Hg exposure (from 0.30 up to 2.40 µg·g^−1^) resulted in major reproductive and behavioural deficits, including reduced offspring independence, less nesting activity, and impaired foraging under stress [36,175,177]. Perkins et al. [198] analysed blood and/or feather samples for Hg concentrations from staging shorebirds from the Yukon area. Mercury exposure and its potential behavioural impacts were examined across several shorebird species. The bar-tailed Godwit (*Limosa lapponica*) showed a Hg concentration of 0.49 µg·g^−1^, though its possible effect on reproductive success could not be evaluated due to missing or potentially biased nesting success data. Among other species, dunlin (*Calidris alpina*) exhibited Hg levels of 0.17 µg·g^−1^, while rock sandpipers (*Calidris ptilocnemis*) showed concentrations of 0.08 and 0.26 µg·g^−1^ in different samples. The semipalmated sandpiper (*Calidris pusilla*) had a concentration of 0.95 µg·g^−1^, the long-billed dowitcher (*Limnodromus scolopaceus*) 0.35 µg·g^−1^, and the red phalarope (*Phalaropus fulicarius*) 0.12 µg·g^−1^ [198]. These values, derived from blood and feather samples collected in the field, provide empirical insights into species-specific sensitivity to Hg exposure, highlighting variability across species that may influence their behavioural and reproductive responses.

### 4.2. Tissue Type and Interpretation of Exposure

The choice of tissue sampled also significantly influences the interpretation of the Hg impact. Blood, as a marker of recent and active exposure, is commonly used in field studies due to its minimally invasive collection (Table 2). Behavioural disruptions in blood-based studies are generally linked to recent or ongoing exposure, as shown in the common loon, the Carolina wren, and the black-legged kittiwake (*Rissa tridactyla*) [22,51,126,199]. For instance, black-legged kittiwakes with blood Hg levels above 1.80 µg·g^−1^ showed increased likelihood of skipping breeding, a behaviour not seen at lower concentrations, suggesting a potential threshold effect [126]. In contrast, feathers, which integrate exposure during their growth, are better suited for assessing long-term or historical exposure. For instance, high feather Hg in the common loon (≥40 µg·g^−1^) was linked to developmental instability, while feather Hg in the grey-faced petrel (*Pterodroma gouldi*; ~39 µg·g^−1^) had no discernible behavioural effects [22,200]. This discrepancy points to the need to contextualise feather Hg results within the species’ ecology and the timing of feather growth. Eggs, on the other hand, serve as direct indicators of maternal transfer and reproductive risk. The consistent finding across several species, including the tree swallow, the Carolina wren, and the lesser black-backed gull, is that egg Hg levels, even at low concentrations (from ~0.07 up to 0.53 µg·g^−1^), can lead to altered incubation behaviour or nest success [184,199,201]. Importantly, the behavioural effects in some studies (e.g., decreased incubation time in tree swallows) occur below physiological thresholds for reproductive failure, reinforcing the sensitivity of behavioural endpoints as early indicators.

### 4.3. Field vs. Laboratory Insights

Another critical distinction is between field and laboratory studies. Field studies, despite capturing ecological complexity, often struggle to establish robust links between Hg levels and behavioural changes due to numerous uncontrollable variables affecting exposure and response. They also show greater variability in outcomes (Table 3). For instance, despite Hg concentrations of 2.77 µg·g^−1^ and 2.16 µg·g^−1^ in feathers, as well as 0.37 µg·g^−1^ in egg yolk, lesser black-backed gulls exhibited no observable behavioural alterations [201]. In contrast, little auks (*Alle alle*) displaying blood Hg between 0.5 and 2 µg·g^−1^ showed detectable physiological stress during diving, including prolonged post-dive recovery times, illustrating the context-dependent nature of sub-lethal effects in natural settings [202]. Laboratory studies, by comparison, benefit from controlled dosing and tend to yield clear dose–response relationships: Consistent findings include reduced reproductive success, altered courtship behaviour, and impaired foraging in species such as the zebra finch and the European starling (*Sturnus vulgaris*) [50,203]. These contrasting outcomes highlight that while Hg can disrupt behaviour at environmentally relevant concentrations, the presence of natural stressors or ecological buffers may mask or modulate such effects in wild populations. Consequently, linking behavioural outcomes to Hg exposure in field settings requires multifactorial approaches that account for variables like age, sex, prey variability, moult status, and seasonal physiology. Combining laboratory and field data, e.g., using bio-logging or isotope analysis to align individual exposure profiles with observed behaviours, can help clarify under what ecological contexts Hg becomes behaviourally significant.

## 5. Considerations and Challenges

**Figure 5:**
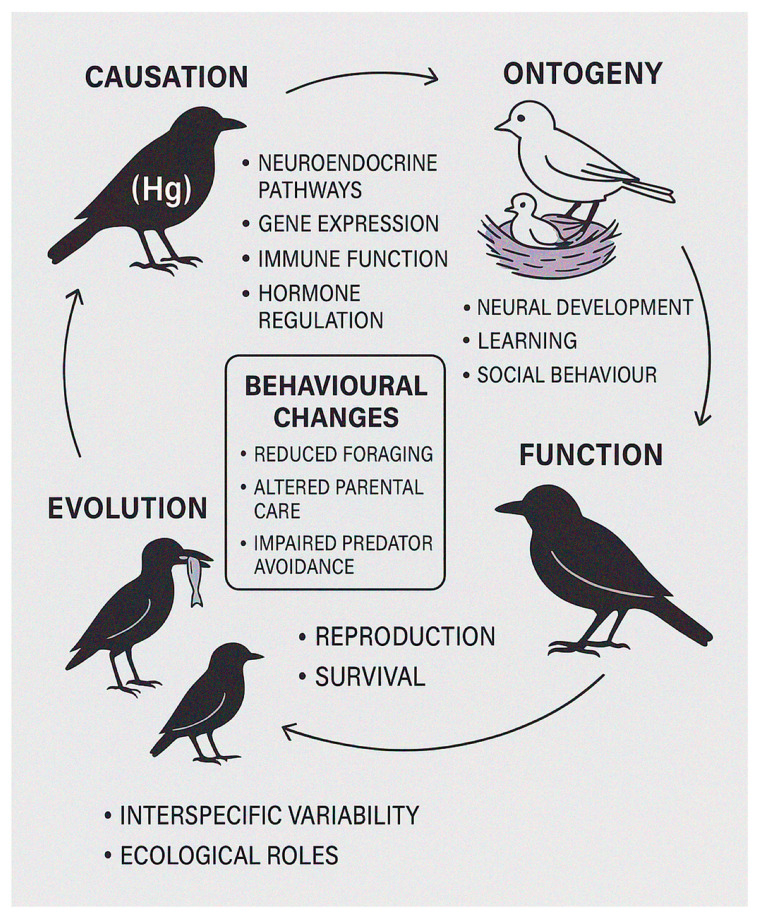
A schematic summary of an integrative framework for understanding behavioural impacts of mercury (Hg) exposure in birds, based on Tinbergen’s four questions: causation, ontogeny, function, and evolution.

Integrating behavioural changes as indicators of toxicological effects in ecotoxicological studies offers the advantage of detecting sublethal changes that traditional laboratory analyses, such as biomarker studies, may miss. Behavioural alterations in birds are often a result of the interaction between multiple environmental stressors, as well as biotic and abiotic factors. These stressors can range from habitat degradation and climate change to direct exposure to pollutants, making behavioural observation a valuable tool for assessing ecological health in the field. Importantly, behavioural changes can occur even at lower levels of exposure that may not induce obvious physiological effects, offering insights into the subtle but significant impacts of environmental pollutants. Laboratory experiments enable controlled investigations of causal relationships and underlying mechanisms, while field studies provide ecological relevance by examining untamed populations in their natural habitats (Figure 5).

Birds serve as valuable bioindicators for monitoring Hg due to their wide distribution, diverse feeding habits, and heightened sensitivity. Species that are particularly suitable for monitoring Hg pollution include those occupying higher trophic levels, such as piscivorous birds that bioaccumulate Hg through their diet. Long-lived species with strong site fidelity, such as the common loon and the white stork, are excellent candidates, as they offer reliable assessments of local pollution over time due to their consistent presence in specific areas. Additionally, the white stork is particularly significant due to its diverse diet, including both aquatic and terrestrial prey, making it a useful indicator of Hg pollution across both aquatic and terrestrial environments. When selecting bird species for Hg studies, it is essential to consider their ecological relevance, habitat preferences, and the availability of baseline data for comparison. Birds that occupy a variety of habitats, from urban to rural, aquatic to terrestrial, can offer a holistic view of Hg pollution. Furthermore, long-term monitoring of a single species or a carefully selected group of species can help identify trends and facilitate comparisons over time, offering deeper insights into the effects of Hg on bird populations.

Although much is known about Hg in birds, key gaps remain, especially in understanding its effects on behaviour. One of the key advantages of using behavioural monitoring is its flexibility in terms of data collection. For long-term studies, birdwatchers and volunteers can be trained to observe and report changes in behaviours over time, creating an extensive and cost-effective database. This makes it possible to monitor large populations of birds over extensive periods without the need for invasive procedures or costly equipment [48]. Additionally, such behavioural data collected in the field can provide a broader and more naturalistic perspective on the impacts of Hg exposure than those derived from controlled laboratory settings.

On the other hand, field-based behavioural observation comes with its own set of challenges. The complexity of natural environments means that birds are exposed to a variety of stressors simultaneously, complicating the attribution of observed behavioural changes to specific pollutant exposures. It can be difficult to differentiate between behavioural changes caused by pollution-related stressors and those resulting from natural environmental fluctuations or other biotic interactions. Indeed, Arctic seabirds, for instance, are not only polluted by MeHg but also by organochlorines and perfluoroalkyl substances (PFAS), among other pollutants. There are interactions between the pollutants’ effects. For instance, in Arctic-breeding adult black-legged kittiwakes from Svalbard, results indicated a negative relationship between the sum of all detected chlordanes and the metabolic rate (MR) in both sexes, whereas perfluorotridecanoate and MRs were positively related in females only. No relationship could be found between MRs and MeHg concentrations [204].

This difficulty highlights the need for a well-established baseline understanding of the species’ ecology and behavioural patterns under natural conditions. For example, the current WHO provisional tolerable weekly intake (PTWI) of 1.60 μg·kg^−1^ body weight·week^−1^ of MeHg has been established as a guideline for people, suggesting exposure up to a certain level is unlikely to cause adverse health effects. However, Bourdineaud et al. [205], in which mice were fed diets mimicking fish consumption, show that when exposure falls below the PTWI, physiological and behavioural changes occur, including altered growth, anxiety, and changes in brain chemistry. These findings suggest that the established thresholds might be insufficient, as they fail to account for the subtle, yet important effects of Hg exposure, particularly on the nervous system and behavioural responses, which could be even more pronounced in species with different metabolic rates, life histories, and ecological exposures. For birds, especially those at higher trophic levels like piscivorous species, the bioaccumulation of Hg through diet may result in even higher exposure levels than those typically assumed by human-centric guidelines. Given that Hg has been shown to affect behavioural responses in avian species, including changes in foraging and migratory behaviours, perhaps the current thresholds might not offer sufficient protection for birds, particularly those with chronic exposure in polluted environments. Therefore, a lower, more precautionary threshold for Hg exposure should be considered, with additional research needed to better understand the cumulative effects of long-term, low-level exposure on avian health and behaviour.

Nevertheless, toxicity reference values have been suggested for birds related to behavioural endpoints. To take just one example, it has been estimated that the effective concentration (EC) of maternal dietary Hg that caused a 5% and 10% reduction in the combined survival and reproduction endpoints for juveniles (EC_5_ and EC_10_) was 0.60 and 1.10 μg·g^−1^ dry weight, respectively [153]. Other authors have summarised the estimated reproductive effect thresholds for birds, and the Hg EC_20_ is in the range of 0.16 to 0.75 and 1.10 mg·kg^−1^ in the diet, giving a range of 2.10 to 4.20 mg·kg^−1^ in the blood [206]. Toxicity benchmarks have also been derived and summarised from the literature and are related mainly to health and physiology, reproduction, and behavioural impairments [2].

Some behavioural changes can be subtle and challenging to quantify, especially when they occur over long timeframes. Behavioural responses to sublethal pollutant exposure often manifest as gradual shifts in activity levels, mating success, or foraging patterns, making it difficult to draw definitive conclusions from short-term observations. Thus, integrating behavioural monitoring into ecotoxicological studies requires a long-term commitment to data collection, sometimes over several years, to account for the natural variability in behaviours and environmental conditions. Another challenge is the lack of standardised thresholds for the concentrations of pollutants, such as Hg, that lead to behavioural changes in birds. While studies have proposed specific concentrations of Hg in tissues (e.g., 5 µg·g^−1^ of dry weight as a threshold for Hg toxicity in feathers [207]), these values are often not universally applicable across different species or habitats. Mercury concentrations that lead to behavioural disruptions in one species may have negligible effects in another, highlighting the necessity for species-specific ecotoxicological thresholds. This variability calls for more targeted research to identify these species-specific thresholds and the ecological conditions under which these thresholds are reached. Moreover, although feather analysis is a promising non-invasive tool for studying Hg exposure in birds, there remains a lack of consensus on how to effectively correlate feather Hg concentrations with specific behavioural outcomes. Ackerman et al. [153] have nevertheless modelled the conversion of feather and tissue Hg concentrations into blood Hg concentrations, providing equations that allow these calculations [153]; conversely, a model giving the Hg concentrations in eggs from the female blood THg has been published [154].

Research has shown that behavioural impacts can occur at lower concentrations of Hg than would result in overt physiological toxicity, suggesting that behavioural monitoring may detect effects before physical symptoms appear [22,208]. To mitigate Hg pollution and protect birds, currently, no widely established or practical large-scale detoxification methods exist to directly reduce Hg pollution in wild bird populations. Strategies such as incorporating Hg chelators into avian diets face significant challenges, including difficulties in delivering these compounds to free-ranging birds, potential ecological side effects, and safety concerns. Consequently, direct detoxification of birds in natural settings is not yet feasible, highlighting the urgent need for further research into safe, effective, and scalable detoxification methods for avian species. Given these constraints, the most effective and practical approach currently available is reducing environmental Hg pollution at its source. Population modelling studies [17] suggest that substantial reductions in Hg emissions could markedly benefit species such as common loons, which experience elevated MeHg exposure. Beyond emission reduction, phytoremediation offers a promising, though still emerging, approach to mitigating Hg pollution in avian habitats. This method employs specific plants and their associated microbial communities to extract, stabilise, or transform Hg in soils and water. Certain phytochemicals have demonstrated potential in chelating heavy metals and promoting microbial degradation of Hg compounds [209]. However, despite encouraging results in controlled or localised environments, scaling phytoremediation to effectively reduce Hg bioavailability across complex and extensive avian habitats presents considerable challenges. Success depends on factors such as the degree and distribution of pollution, plant species selection, ecological interactions, and sufficient time for remediation to take effect.

### Recommendations for Future Research

In light of the identified challenges and knowledge gaps, the following specific recommendations are proposed to guide future research on the behavioural impacts of Hg in birds:

- Mechanistic studies at the molecular and neuroendocrine levels: there is a critical need for research elucidating the molecular, neurological, and endocrine pathways through which Hg disrupts avian behaviour. Understanding these mechanisms will improve our ability to link behavioural symptoms with specific biochemical effects and dose-response relationships.
- Long-term behavioural monitoring in natural populations: extended field-based studies are essential to capture subtle and cumulative behavioural effects that develop over time. Such studies should be designed to span multiple breeding seasons and account for natural variability in behaviour and environmental conditions.
- Species-specific toxicity thresholds: due to interspecific differences in sensitivity and exposure routes, future work must aim to define species-specific ecotoxicological thresholds for both physiological and behavioural endpoints. This includes establishing reliable reference values for tissue Hg concentrations and their behavioural consequences.
- Standardisation of behavioural endpoints in ecotoxicology: to integrate behavioural changes more effectively into ecotoxicological frameworks, standard protocols and validated metrics must be developed. Harmonising laboratory and field approaches will allow for stronger causal inferences and more ecologically meaningful assessments.
- Advancement of non-invasive biomonitoring techniques: refining the use of feathers, blood, and egg samples to assess Hg exposure requires improved calibration against known behavioural outcomes. Validated models converting Hg concentrations across tissue types (e.g., feather to blood, blood to egg) should be expanded across species and ecological contexts.
- Assessment of pollutant interactions: future studies should examine how Hg interacts with other pollutants, such as organochlorines and PFAS, in shaping behavioural outcomes. Synergistic, antagonistic, or amplification effects may obscure Hg toxicity, necessitating multi-pollutant risk assessments.
- Scalable detoxification and mitigation strategies: research into practical and ecologically sound detoxification strategies. Studies must assess their safety, effectiveness, scalability, and potential ecological consequences in real-world avian habitats.
- Population-level modelling and risk assessment: continued development of population models will help forecast demographic responses to changes in Hg exposure and evaluate the conservation benefits of mitigation efforts, including reductions in global Hg emissions.
- Citizen science and community engagement: incorporating trained volunteers and birdwatchers into long-term behavioural monitoring programs offers a cost-effective means of collecting large-scale data. Citizen science initiatives should be carefully structured to ensure data reliability and enhance public awareness of Hg ecological impacts.

## 6. Conclusions

This review demonstrates that behavioural alterations represent a sensitive and ecologically meaningful consequence of Hg exposure in birds, with evidence spanning mechanistic disruptions, developmental trajectories, functional outcomes, and evolutionary contexts. Mercury affects critical behaviours, such as foraging, parental care, predator avoidance, and social communication at environmentally relevant concentrations, often preceding observable physiological or reproductive impairments. These behavioural disruptions are mediated by complex neuroendocrine and immunological pathways, many of which remain insufficiently characterised, highlighting a need for mechanistic research at the molecular level. Ontogenetic effects show that early-life Hg exposure induces persistent behavioural deficits, underscoring the importance of developmental timing in toxicological assessments. Functionally, these behavioural impairments reduce individual fitness by compromising reproductive success and survival capabilities. Comparative analyses reveal considerable interspecific variability in both Hg accumulation and behavioural sensitivity, particularly in species occupying higher trophic levels or specialised ecological niches. This heterogeneity stresses the necessity of species-specific ecotoxicological thresholds and tailored conservation strategies. Methodologically, the integration of behavioural endpoints into ecotoxicological frameworks presents both an opportunity and a challenge. Behavioural data offer a non-invasive and early indicator of sublethal toxicity, yet require rigorous baseline knowledge of species ecology, standardised metrics, and long-term monitoring to account for natural behavioural variability. Field and laboratory studies, when combined, provide a comprehensive perspective. Field studies capture ecological reality, while laboratory experiments yield causal inference. The behavioural effects of Hg in birds are neither incidental nor secondary; they are central to understanding the ecological consequences. Future ecotoxicological research and environmental policy must prioritise behaviour as a core endpoint to improve the detection, interpretation, and mitigation of pollutant impacts on avian populations and biodiversity at large.

Reducing global Hg pollution is therefore essential not only for avian health but also for the protection of entire ecosystems. Effective mitigation strategies include stricter enforcement of international agreements such as the Minamata Convention, which aims to limit anthropogenic Hg emissions through controls on industrial processes, waste management, and product manufacturing. Transitioning to cleaner energy sources, particularly phasing out coal-fired power plants, represents a critical step toward reducing atmospheric deposition of mercury. Additionally, promoting sustainable ASGM practices through education, alternative technologies, and financial incentives can substantially decrease Hg release in developing regions. Restoring wetlands and riparian buffers can help sequester Hg in sediments, thereby limiting its bioavailability to wildlife. Continued global monitoring and public health outreach are also vital for ensuring accountability and raising awareness of Hg ecological risks. Together, these measures can support a proactive, multi-scale response to curbing Hg pollution and safeguarding behavioural integrity in birds and beyond.

## Figures and Tables

**Table 1:** Species-specific sensitivity to mercury (Hg) exposure in birds. Summary of studies highlighting differences in behavioural responses among avian species at various Hg concentrations.

Species	Study Type	Tissue	Hg Concentration (µg·g^−1^)	Observed Effect	Reference
Common loon, *Gavia immer*	Field	Blood	3.00	Increased lethargy, reduced incubation time	Evers et al. [22]
Mallard, *Anas platyrhynchos*	Lab	Dietary	0.50	Reduced clutch size, altered behaviour	Heinz [167]
Zebra finch, *Taeniopygia guttata*	Lab	Dietary	0.30–2.40	Reduced reproductive success, impaired foraging	Varian-Ramos et al. [177]
Tree swallow, *Tachycineta bicolor*	Field	Blood/Egg	0.07–3.03	Reduced fledging success, altered incubation	Hallinger and Cristol [197], Hartman et al. [184]

**Table 2:** Influence of tissue type on mercury (Hg) exposure assessment and behavioural effects. Overview of studies emphasising the role of blood, feathers, and eggs in interpreting Hg levels and associated behavioural outcomes.

Species	Study Type	Tissue	Hg Concentration (µg·g^−1^)	Observed Effect	Reference
Common loon, *Gavia immer*	Field	Feathers	≥40	Developmental instability (asymmetrical feathers)	Evers et al. [22]
Black-legged kittiwake, *Rissa tridactyla*	Field	Blood	1.80	Increased likelihood of skipping breeding	Tartu et al. [51]
Tree swallow, *Tachycineta bicolor*	Field	Eggs	0.07–0.53	Altered incubation behaviour, reduced success	Hartman et al. [184,199]
Carolina wren, *Thryothorus ludovicianus*	Field	Blood/Feathers/ Eggs	0.70–3.00 (blood, feathers), 0.11 (eggs)	10% reduction in nest success	Jackson et al. [199]
Grey-faced petrel, *Pterodroma gouldi*	Field	Feathers	38.2–39.50	No significant effect on breeding outcomes	Rewi et al. [200]

**Table 3:** Comparison of field and laboratory studies on mercury (Hg) behavioural impacts in birds. Summary contrasting findings from field observations and controlled laboratory experiments, illustrating variability in Hg effects under different conditions.

Species	Study Type	Tissue	Hg Concentration (µg·g^−1^)	Observed Effect	Reference
Lesser black-backed gull, *Larus fuscus*	Field	Egg Yolk, Feathers	0.37–2.77	No observable behavioural effects	Santos et al. [201]
Little auk, *Alle alle*	Field	Blood	0.50–2.00	Prolonged post-dive recovery (physiological stress)	Grunst et al. [202]
Zebra finch, *Taeniopygia guttata*	Lab	Dietary	0.30–2.40	Reduced reproductive success, impaired foraging	Varian-Ramos et al. [177]
European starling, *Sturnus vulgaris*	Lab	Dietary	0.75–1.50	Reduced flight escape response, increased moulting	Carlson et al. [203]

## Data Availability

No new data were created or analysed in this study.
